# Case report: A case with atypical presentation oftesticular choriocarcinoma

**DOI:** 10.3389/fonc.2024.1223873

**Published:** 2024-07-17

**Authors:** Bin Zhong, Tao Zhang, Yi Dong, Wei Yin, Jian-Xin Zhang, Wei-Dong Jin

**Affiliations:** ^1^ The Sixth Affiliated Hospital, Sun Yat-Sen University, Guangzhou, China; ^2^ Department of Emergency, Renmin Hospital of Wuhan University, Wuhan, China; ^3^ Department of General Surgery, General Hospital of Central Theater Command, Wuhan, China

**Keywords:** missing diagnosis, retroperitoneal mass, testicular choriocarcinoma, case report, preoperative test

## Abstract

Testicular choriocarcinoma is a relatively rare malignancy with a highly aggressive nature. Timely diagnosis and treatment can help prolong the survival of patients and even cure them. This case reports a 29-year-old male who presented to the clinic for a month with epigastric pain. On examination, a massive mass of approximately 9*10 cm could be palpated in the upper abdomen. When asked about his previous history, the patient only described a history of a right inguinal hernia that had been repaired 12 years earlier. The admission diagnosis was considered the retroperitoneal tumor, which was found to have metastasized to the liver and lungs after the completion of relevant tests. We then performed a CT-guided lune puncture biopsy on day 8 of admission. The biopsy pathology suggested metastatic cancer was considered. As the symptoms of tumor compression gradually worsened, we performed surgical treatment (retroperitoneal tumor resection + partial duodenal resection + enteroanastomosis) on day 13 of admission. The postoperative pathology was choriocarcinoma. We subsequently conducted a detailed inquiry with the patient’s family about his medical history and found a history of inguinal testicle. Through testicular ultrasound examination, it was preliminarily determined to be testicular choriocarcinoma (not yet pathologically confirmed). We wanted to start salvage chemotherapy as soon as possible after surgery. However, the patient’s postoperative condition was poor, with rapid progression of hepatopulmonary metastases and gradually increased thyrotoxicosis, and we started salvage chemotherapy (EP regimen: etoposide and cisplatin) on postoperative day 12. However, the patient was forced to stop due to a severe chemotherapy reaction and died of respiratory and cardiac arrest in the hospital. For male patients with retroperitoneal mass, the possibility of germ-cell neoplasm should first be excluded. By inquiring in detail about a history of cryptorchidism and in the initial days of hospitalization, testicular exploration, ultrasounds, and serum tumor markers (AFP, β-HCG) tests can be conducted to rule out the possibility of germ-cell neoplasm, thereby preventing misdiagnosis and treatment delays. If the clinical diagnosis is metastatic germ-cell tumor with severe symptoms of metastatic disease, surgery should never be used as the initial treatment.

## 1 Introduction

Testicular cancer (TC) is a rare disease whose incidence and mortality have increased in recent years (1). Germ-cell tumors account for approximately 95% of TC, including both semi−nomas and non−seminomatous germ cell tumors (NSGCT). NSGCT comprises different histological subtypes, including embryonal carcinoma, yolk sac carcinoma, teratoma, choriocarcinoma, and mixed testicular choriocarcinoma. Of these, testicular choriocarcinoma is the rarest, accounting for less than 1% of germ cell tumors (2). In the United States, the 5-year survival rate for testicular choriocarcinoma is less than 80%, reflecting the invasiveness and pertinacity of the disease (3).

Notably, choriocarcinoma is a curable and chemosensitive disease, but treatment of testicular choriocarcinoma remains a difficult challenge due to its greater resistance to conventional chemotherapy relative to other kinds of choriocarcinoma. Clinical diagnosis should be followed promptly by radical orchiectomy to properly diagnose and control the primary tumor. Subsequently, chemotherapy plays a crucial role in either early or advanced stages (4). However, treatment options for refractory or recurrent patients are limited. In such cases, alternative chemotherapy regimens and high-dose chemotherapy combined with autologous stem cell transplantation may be considered (5). Testicular choriocarcinoma presents with diverse clinical manifestations, and the sites of its metastases are also varied. For an accurate diagnosis of testicular choriocarcinoma, physicians must possess a strong sense of differential diagnosis and utilize CT-MRI, histopathology, and blood biochemical markers for confirmation (6, 7).

Testicular choriocarcinoma occurs mainly in young men (25y-30y). Typical clinical features include high hormone levels (β-HCG) due to the tumor and/or the related symptoms of metastatic diseases, such as compression and bleeding. Signs may include gynecomastia, testicular swelling, etc. (8). However, in patients with atypical symptoms, especially in a subset of patients with retroperitoneal tumor and extensive metastases, the rarity of the disease combined with the highly aggressive nature of choriocarcinoma makes it easy for clinicians to miss the diagnosis and delay treatment thereby increasing patient mortality and leading to tragic events. The purpose of this case report is to review a case of missed testicular choriocarcinoma at our center and to provide a cautionary tale for managing patients with atypical presentation of testicular choriocarcinoma.

## 2 Case report

**Figure 1:**
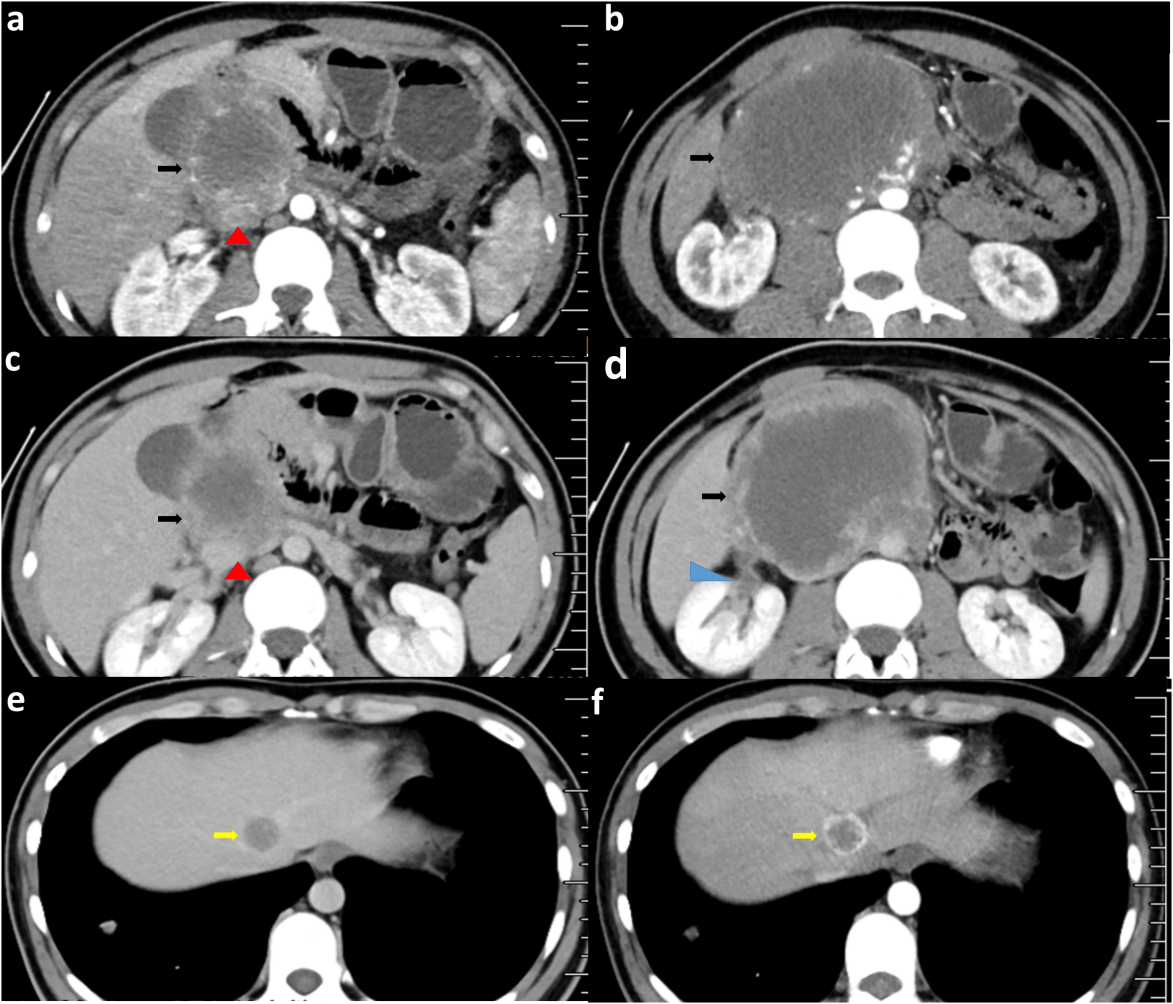
CTA of the abdominal aorta at patient admission. **(A, B, F)** show artery-phase images. **(C, D, E)** are venous-phase images. The black arrow points to the tumor; the red triangle points to the inferior vena cava (the boundary between the inferior vena cava and the tumor is unclear); the blue arrow suggests renal pelvis expansion changes. The yellow arrow points to the liver metastasis.

**Figure 2:**
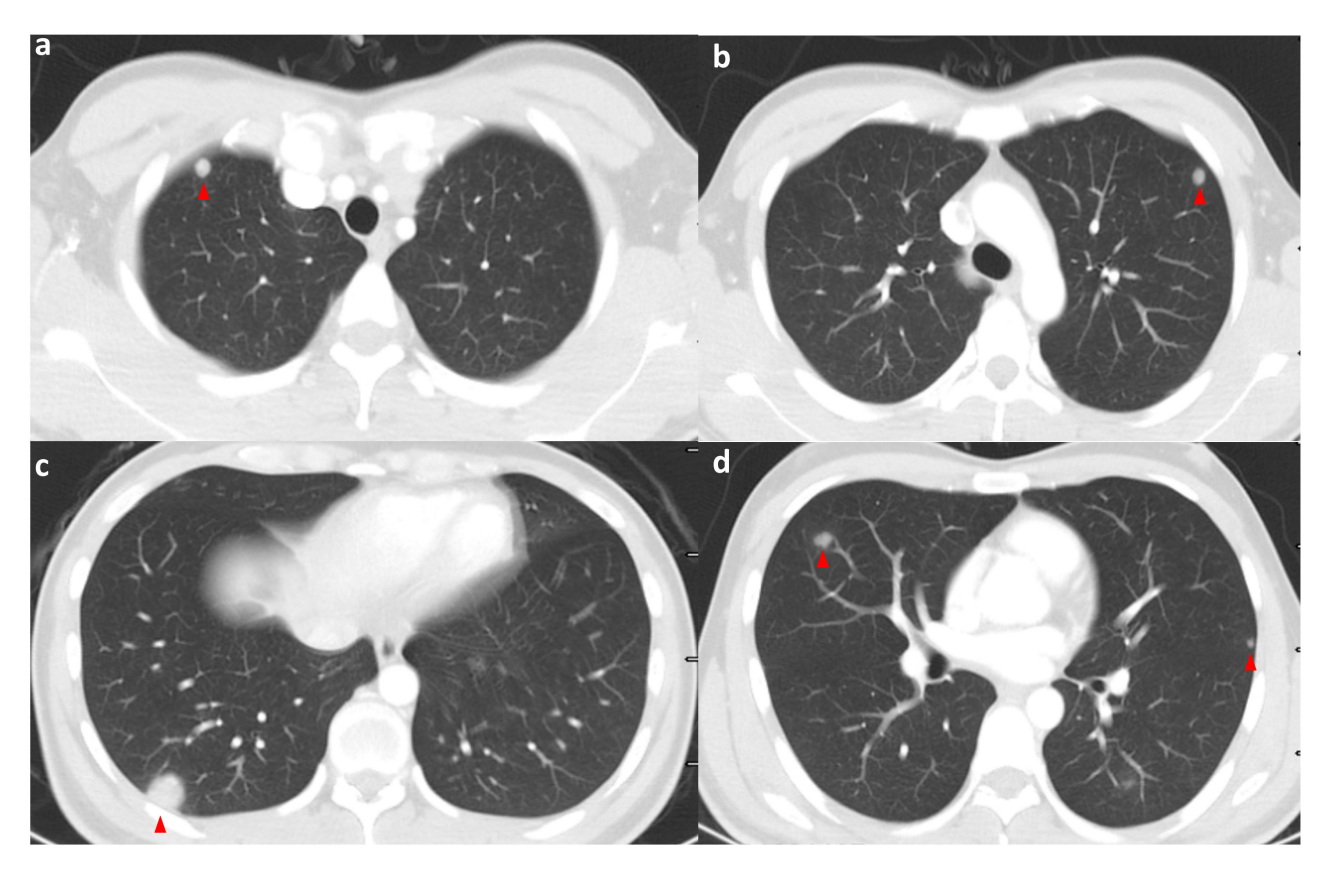
High-resolution CT of the patient on admission (pulmonary window); the patient’s lungs are seen to have multiple pulmonary metastases on admission. The red triangle points to the met. **(A–D)** shows high-resolution CT (lung window) images of different levels of the patient's lungs at the time of admission, where multiple pulmonary metastases were visible. Red triangles point to the metastases.

**Figure 3:**
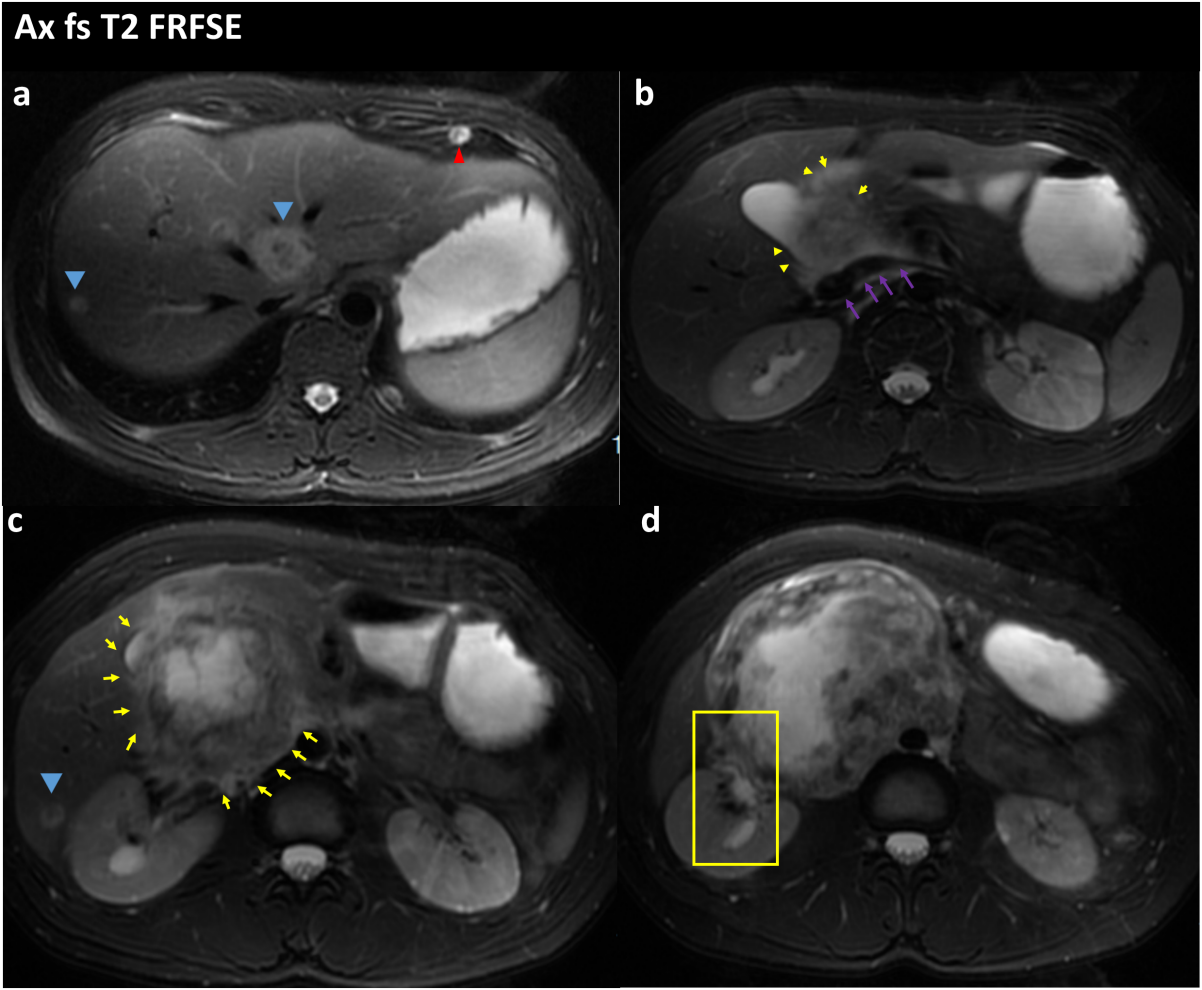
FS-T2WI image of the patient’s abdominal MRI on admission; the yellow arrow points to the location of the abdominal tumor; the blue arrow points to the liver metastases; the red arrow points to the lung metastases; the purple arrow points to the location of the inferior vena cava; **(A)** Tumor with multiple liver and lung metastases. **(B)** the tumor is seen encircling the inferior vena cava and is poorly demarcated from the inferior vena cava. **(C)** Tumor is in the form of an irregular lump, with internal heterogeneous enhancement and blurred margins. **(D)** the right renal pelvis is seen in the yellow box and is tortuously dilated and poorly demarcated from the tumor.

A 29-year-old Asian male was admitted to the hospital with progressively increasing epigastric pain for one month and a weight loss of 5 kg over the past month. When asked about his medical history, he said he had undergone inguinal herniorrhaphy for a right inguinal hernia 12 years ago. There was no history of exposure to radiation or toxins. On admission, a 9*10 cm swelling in the upper abdomen was palpable. There was no significant gynecomastia or visual abnormality of the testes or penis. All vital signs were within normal limits. Only the white blood cell count (11.5*10^9) and neutrophil percentage (84.6%) were high on the first laboratory examination. There were no abnormalities in liver function, kidney function, electrolytes, and serum tumor markers relating to the gastrointestinal and hepatobiliary tract (CEA, AFP, and CA19-9). We performed a preoperative imaging evaluation 5 days after admission. Computed tomographic angiography (CTA) of the abdominal aorta suggested: 1. a right retroperitoneal occupying lesion (size approx. 11.3cm*10.7cm), considered malignant neoplastic (malignant stromal tumor? (suspected duodenal origin); the adjacent inferior vena cava was compressed and narrowed an unclear boundary with the tumor. 2. Multiple abnormal enhancing lesions in the liver, considering metastases (**Figure 1**, three-dimensional reconstructions have been attached to the **Supplementary Material**). High-resolution CT suggests multiple enhancing nodules in both lungs, considering multiple metastases in both lungs (**Figure 2**). Magnetic Resonance Imaging (MRI) suggests 1. a malignant lesion in the right abdominal cavity with liver and multiple metastases in both lungs, 2. right ureter compression, right renal obstructive hydronephrosis, and multiple lymph nodes enlargement around the lesion (**Figure 3**). After the examination, the diagnosis was considered retroperitoneal malignancy combined with hepatopulmonary metastases.

**Figure 4:**
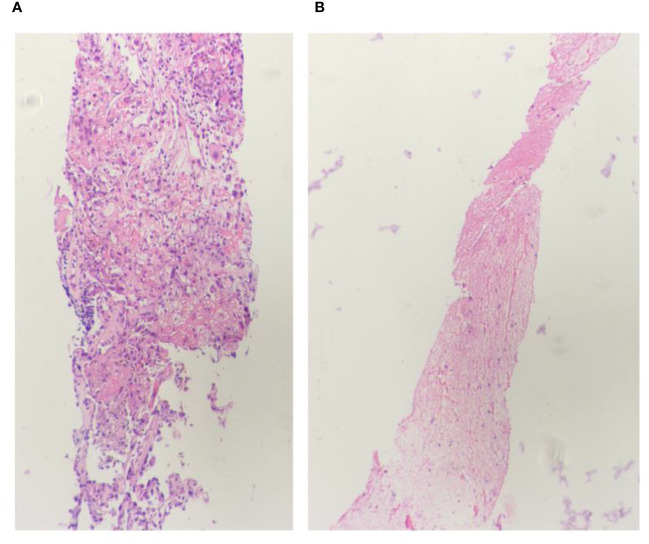
Lung biopsy 20x light microscope image. **(A, B)** respectively shows 20x light microscope images of two lung biopsy tissues.

**Figure 5:**
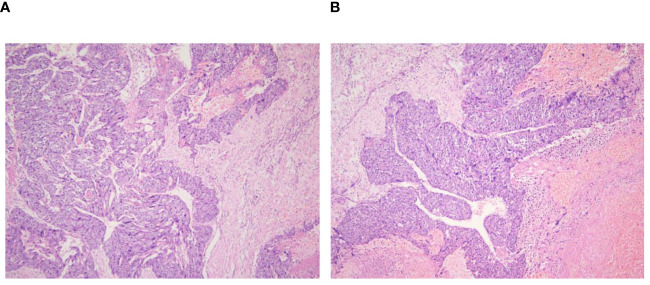
Retroperitoneal tumor pathology 40x light microscope image. **(A, B)** respectively shows 40× light microscope images of retroperitoneal tumor pathology at different locations.

Due to the patient’s distant metastases, radical surgery was impossible. We wanted to obtain tumor pathology to guide the next treatment. In order to rule out a tumor of gastrointestinal origin, we had a gastroscopy on the 7th day of admission, and the results suggested a submucosal bulge in the descending duodenum, which was considered a pressure change. The next day, we performed a CT-guided lung tissue puncture biopsy. The pathology revealed a large area of hemorrhagic necrosis and normal lung tissue structure, with a few clusters of atypical cells, combined with immunohistochemical findings to diagnose metastatic carcinoma (**Figures 4A, B**). During the refinement of the examination, the patient’s epigastric pain gradually increased, and he had severe insomnia at night with severe nausea and vomiting. At the same time, the heart rate gradually increased (the preoperative average heart rate reached 120 beats per minute). The patient strongly desired surgical treatment to relieve his increasing clinical symptoms. At that time, we considered that the onset of these symptoms could be related to a large retroperitoneal lesion compressing the duodenum and the inferior vena cava. The decision to operate was made after the surgical team meeting because the occupying effect can be reduced and pathology can be clarified so that comprehensive treatment can be started as soon as possible. The surgical treatment (retroperitoneal tumor resection + partial duodenal resection + enteroanastomosis) was performed on the 13th day of admission. The patient underwent an uneventful surgical procedure, which lasted six h. The intraoperative bleeding was approximately 1000 ml, and 1200 ml of suspended red blood cells were infused. Postoperative symptomatic and supportive treatment include anti-infection, prevent vomiting, analgesia, fluid infusion, etc. The postoperative pathology described that microscopic view of cancer tissue with extensive hemorrhage and necrosis, and in conjunction with the clinic and immunohistochemical results, choriocarcinoma was considered. Immunohistochemistry: Pan-cytokeratin, CK7, CK19 (+), CD10 (+/-), HepPar1, TTF1, CD34, Syn, Gly-3, EMA, Villin, PLAP, CD30, AFP, OCT4, CD117 (-), GS (glutamine synthetase) focal slight (+), β-HCG (+), SALL4 focally (+), KI-67: 80% (**Figures 5A, B**).

**Figure 6:**
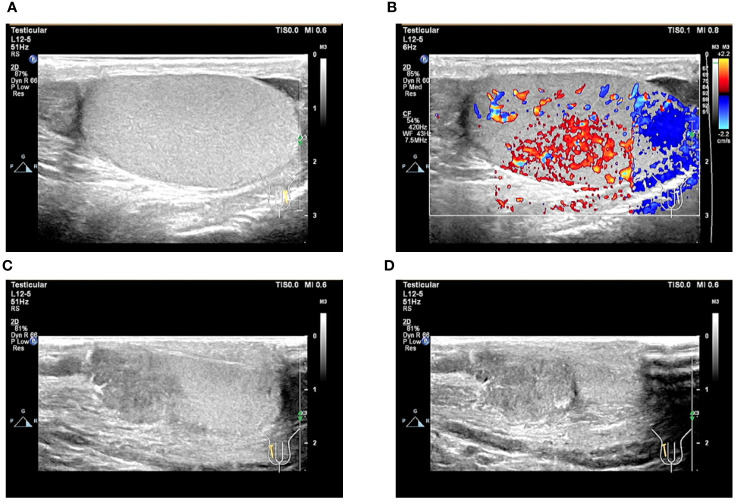
Ultrasound image of the testis. **(A, B)** Ultrasound image of the left testis; **(C, D)** Ultrasound image of the right testis.

After obtaining the pathological results, we asked the patient’s parents for a detailed history and learned that the patient had a history of inguinal testicle. The patient had undergone a right inguinal hernia repair and testicular descent fixation at the same time 12 years ago, and due to the young age of the patient at that time, he was unaware of his history of inguinal testicle. On palpation of the testis, the right testis was found to have no apparent substantial mass but was poorly mobile and surface not smooth. Blood HCG test >10,000mIU/mL. Testicular ultrasound was performed, and the findings suggested the right testicle is morphological abnormalities. The heterogeneous echo of the parenchyma. Several solid nodules were seen within the parenchyma (**Figures 6A–D**). The diagnosis of testicular choriocarcinoma with liver, lung, and retroperitoneal metastases was basically confirmed. The patient’s heart rate dropped to an average of 80 beats/min within three days after surgery but gradually increased to 130 beats/min by the 7th postoperative day, and his general condition was deteriorating. For example, the patient showed no obvious signs of bleeding, but the hemoglobin was still dropping slowly. Insomnia increased at night, and the basal metabolic rate [(pulse rate + pulse pressure) -111] was above 40%.

**Figure 7:**
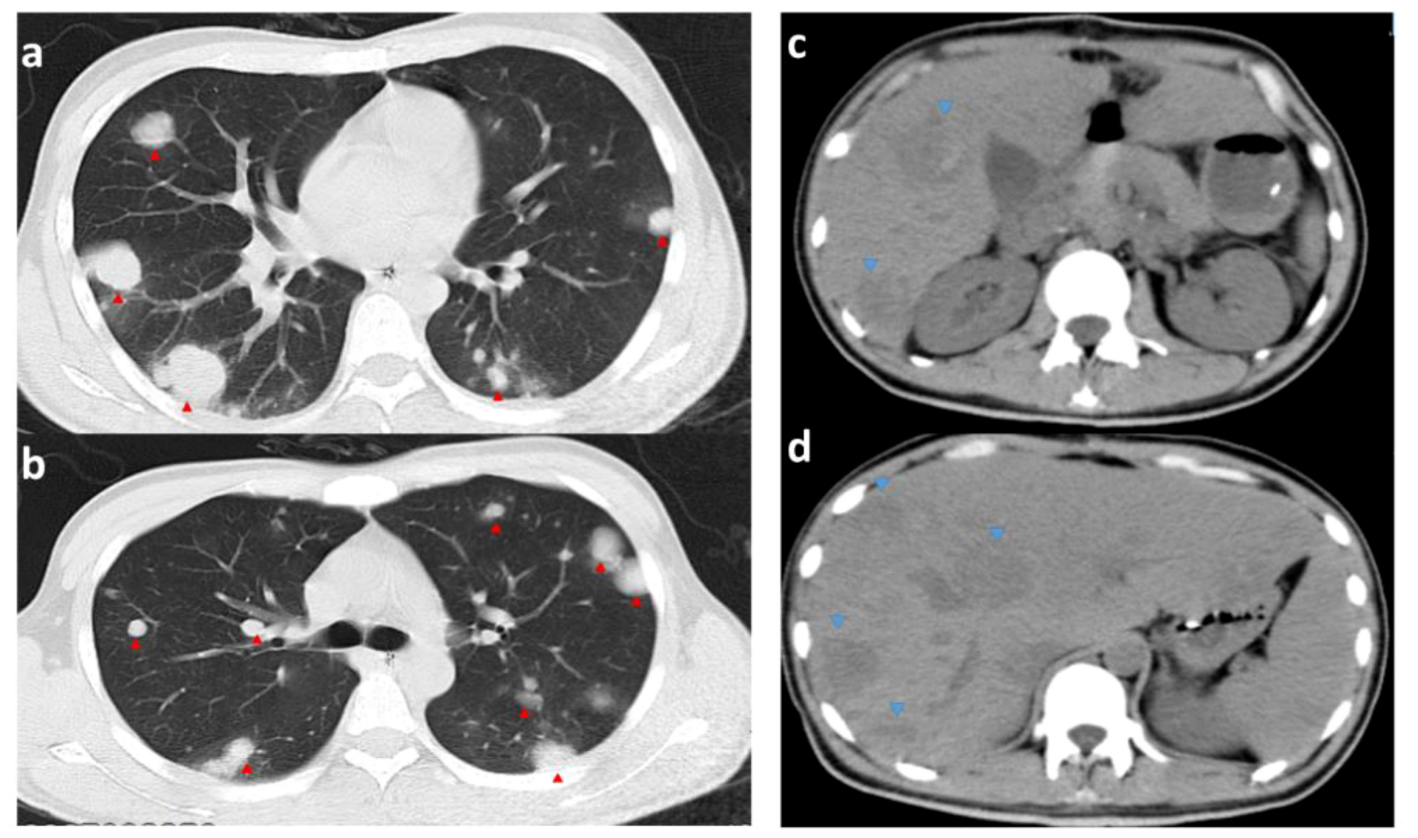
CT images of the chest and abdomen on the 6th postoperative day. **(A, B)** are CTs of the chest (pulmonary window); **(C, D)** are CTs of the abdomen; red arrows point to metastases in the lungs (significantly more extensive and numerous than before surgery). Blue arrows point to liver metastases (hypointense shadow significantly larger than before surgery).

When the pathology results were available, thyroid function was urgently checked: T3, FT3, T4, and FT4 were all significantly elevated. Propranolol (20 mg orally 4/day) was given to control the heart rate, but the mean heart rate was still high (mean 120 beats/min). The 6th postoperative CT of the chest and abdomen showed rapid progression of liver and lung metastases (**Figure 7**). To improve the patient’s survival, we wanted to start salvaging chemotherapy as a matter of urgency. Considering the patient’s lung tumor load, we used the EP regimen (etoposide and cisplatin) without bleomycin to avoid accentuating the respiratory compromise. We started the first chemotherapy on postoperative day 12 but were forced to stop as the patient developed a severe coughing up of blood. The patient did not undergo an orchiectomy. He eventually died of respiratory and cardiac arrest on postoperative day 14.

## 3 Discussion

Testicular choriocarcinoma is a specific type of testicular cancer representing a subtype of NSGCT. It often presents with high levels of HCG, is found with extensive metastases, has rapidly progressive disease, is poorer sensitivity to chemotherapy than other choriocarcinomas, and has an inferior prognosis (9). Diagnosing early and treating can prolong the patient’s survival. The typical clinical manifestations of the disease include testicular masses, gynecomastia, elevated serum HCG, multiple metastatic lesions, anemia, and hyperthyroidism (10). However, it often presents with hemorrhage and pressure symptoms at the metastatic site rather than a palpable testicular mass. The most common sites of metastasis for testicular choriocarcinoma are the lungs and brain, often with hemoptysis as the initial presentation (2). More commonly, the disease is not diagnosed until advanced stages. Laboratory investigation involves tumor markers such as β-HCG and AFP. β-HCG is significantly high in pure choriocarcinoma (> 1000 IU/L, normal range is < 2 mIU/mL) and choriocarcinoma does not produce AFP (11). The standard radiologic test for testicular choriocarcinoma is ultrasound. Choriocarcinoma usually presents as a well-defined hypoechoic lesion with areas of hemorrhage and necrosis and may show more in-homogenous cystic appearance than other types of testicular cancer. In conjunction with physical examination, ultrasound sensitivity can be 100%. CT is necessary to determine the extent of metastasis for staging purposes (12).

In this report, we detail the patient’s history from admission to death and the relevant imaging data, hoping that clinicians can learn from this experience and each other. It is important to note that in cases where the diagnosis of a retroperitoneal tumor is unknown, we should consider the diagnosis in detail preoperatively and rule out a germ-cell neoplasm first. Add screening for tumor markers or perform simple tests for differential diagnosis as appropriate. In this case, the urine pregnancy test was almost undoubtedly positive and could have been considered this disease if we had performed a urine pregnancy test after the patient was admitted to the hospital. Meanwhile, if the patient had been screened with testicular ultrasound or PET-CT on admission, this might not have led to such an error. Of course, a lack of knowledge of rare diseases is a common reason for clinicians to miss a diagnosis, especially if it is outside their expertise. When the diagnosis is unclear, a multidisciplinary consultation can be considered to reduce the rate of underdiagnosis. We recommend that patients presenting with a retroperitoneal mass as the primary symptom should routinely undergo a genital ultrasound to screen for the source. The chance of asymptomatic brain metastases is high in patients with high HCG levels. Therefore, most patients undergo brain MRI to complete the disease staging. The PET-CT can be performed if available, PET-CT can be performed to help determine primary and metastatic lesions and also to help prevent complications related to metastases. For example, if a patient has metastases in the skull, the risk of intracranial hemorrhage can be assessed. A literature review found that excessive human chorionic gonadotropin might lead to thyrotoxicosis, which produces a range of clinical signs of high BMR (13–15).

Choriocarcinoma is one of the malignancies that can be cured by chemotherapy. Serum HCG levels are often indicative of tumor activity as well as tumor burden (16).

In general, testicular germ-cell tumor is a malignancy with good therapeutic potential, even in advanced stages (17). However, testicular choriocarcinoma is a specific type of germ-cell tumor that is often stubbornly resistant to chemotherapy, and some patients develop refractory or progressive disease despite high-dose remedial chemotherapy. Failure to achieve complete tumor remission after multiple remedial treatments is a clear indicator of poor prognosis in this particular patient population (2). Typically, radical orchiectomy should be performed after a clinically confirmed diagnosis of testicular choriocarcinoma to allow for proper diagnosis and primary tumor control (18). Timely initiation of chemotherapy is also a determinant of prognosis in metastatic testicular choriocarcinoma, and first-line chemotherapy is recommended with cisplatin-based regimens, including EP, bleomycin EP, oretoposide, ifosfamide and cisplatin (VIP). Patients with testicular choriocarcinoma should receive at least four cycles of chemotherapy and continuous monitoring of HCG (2). In addition, for patients who have relapsed or developed resistance to first-line chemotherapy, combination salvage chemotherapy such as paclitaxel, ifosfamide and cisplatin (TIP), gemcitabine and oxaliplatin (GemOx), gemcitabine, oxaliplatin and pacli− taxel (GOP), or irinotecan combined with nedaplatin is usually recommended (19, 20).

This patient’s serum HCG was above the upper line after surgery, indicating a severe tumor load and a poor prognosis. Meanwhile, in the present case, the patient had a normal-sized testis but presented with a large abdominal mass as the primary symptom, with hepatopulmonary metastases. The literature has reported that the rare simple choriocarcinoma exhibiting hepatopulmonary metastasis features with normal testicular morphology is the most aggressive and has an abysmal prognosis (21). Such patients should be treated with urgent chemotherapy to improve survival when markedly elevated AFP or β-HCG levels in an appropriate context prompt the start of chemotherapy urgently (available chemotherapy regimens are: EP, BEP, VIP, etc.) even without the need of biopsy confirmation. But the best time to start chemotherapy in postoperative patients is uncertain, the optimal timing of chemotherapy for such cases needs to be further explored.

It is worth noting that the rapid progression of hepatopulmonary metastases in patients after resectioning a sizeable retroperitoneal mass may be associated with the metastases becoming more aggressive after reducing the tumor load. This also alerts us to the fact that if a clinical diagnosis of metastatic germ-cell tumor is made with severe symptoms of metastatic disease, surgery should never be used as initial treatment. In addition, guidelines suggest that testicular cancer may be more aggressive when it metastasizes, so a biopsy of metastases is not recommended (22). Whether reducing the tumor load also leads to a similar transformation needs further exploration. Another topic for discussion is the choice of chemotherapy regimen. Because each patient’s general condition and tumor load are different, individualized treatment regimens should be given to achieve optimal results while reducing the side effects of chemotherapy. The typical treatment regimen for testicular choriocarcinoma is the BEP regimen (bleomycin, etoposide, and cisplatin), for which most patients have been reported to tolerate well and achieve high-quality remission with a controlled total HCG level of 600 IU/L at six-month follow-up (23). Papiani et al. (24) used high-dose carboplatin in combination with etoposide in conjunction with autologous peripheral blood stem cell transplantation to treat simple male choriocarcinoma and achieved good results. In this patient, we used the EP regimen, as the patient was considered to have a severe postoperative lung tumor load to avoid potential respiratory failure resulting from bleomycin. Meanwhile, the high risk of pulmonary hemorrhage should also be considered, and the dose of chemotherapy is usually kept low (baby EP protocol). However, due to choriocarcinoma’s aggressive nature, we propose adding bleomycin to the subsequent chemotherapy cycles. Unfortunately, the patient experienced a severe gastrointestinal reaction on the first day of chemotherapy and was forced to stop. The small number of patients with this type of disease makes clinical research tricky. As mentioned above, control of advanced choriocarcinoma has been reported with non-standard therapies. It is hoped that there will be more possibilities for treating male testicular choriocarcinoma patients, which will require continued basic and translational research.

With the development of science, technology, and the economy, standard screening equipment is available in some basic hospitals. The missed diagnosis rate of common diseases by clinicians has gradually decreased, but the rate of rare diseases is still high. One reason may be that clinicians rely too heavily on equipment and neglect to improve their diagnostic skills. Secondly, with the increasing specialization, doctors are losing sight of knowledge not part of their specialty. Machines cannot replace the role of human beings, and it is an essential requirement for doctors in the new era to improve their abilities and constantly enrich their knowledge base. This is to be responsible for each patient and to safeguard one’s medical career.

## 4 Conclusion

In practice, clinicians can easily miss the diagnosis of rare diseases, especially when the disease has no typical symptoms. This situation often leads to delayed treatment and potentially severe consequences. We aim to review this case as a reminder that patients with retroperitoneal masses should constantly be screened for possible reproductive system tumors (either from physical examination, tests, or examinations) and, if necessary, for multidisciplinary consultation. If multiple distant metastases accompany the retroperitoneal tumor, conditions allow PET-CT examination to help to judge metastases and primary lesions to avoid missed diagnosis.

## Data availability statement

The original contributions presented in the study are included in the article/**Supplementary Material**, further inquiries can be directed to the corresponding author/s.

## Ethics statement

Written informed consent was obtained from the individual(s) for the publication of any potentially identifiable images or data included in this article.

## Author contributions

BZ and TZ helped in writing the abstract, figure collections, writing, and format. YD helped in writing and format. WY helped in writing and editing. J-XZ and W-DJ helped in review and final editing. All authors contributed to the article and approved the submitted version.
