# Supplementary figures and images for: Case report: A case with atypical presentation oftesticular choriocarcinoma
Source: Front Oncol. 2024 Jul 17;14:1223873. doi: 10.3389/fonc.2024.1223873 (PMC11294831; doi:10.3389/fonc.2024.1223873)

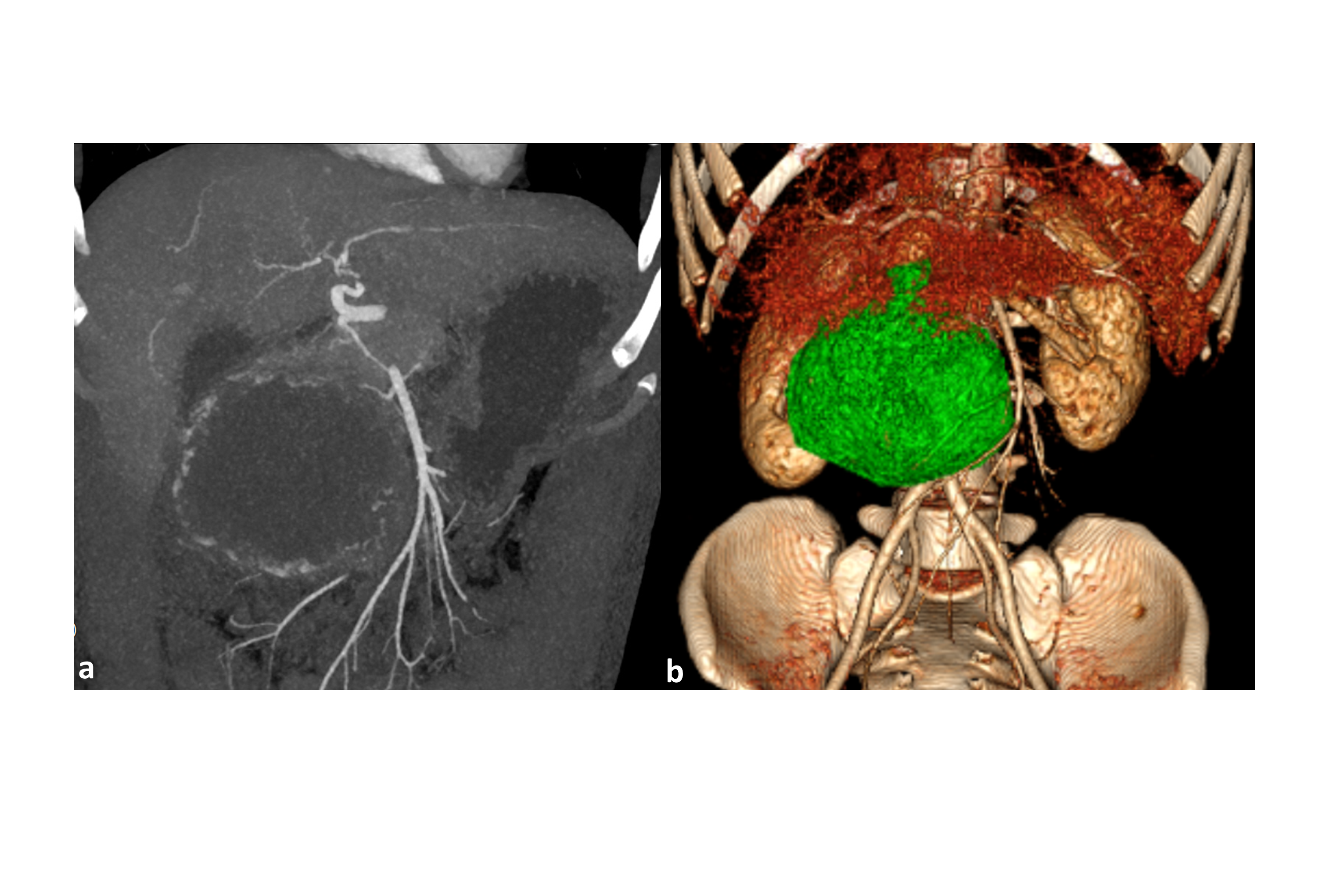

Supplement: Supplementary file 1 [file Image_1.tif]

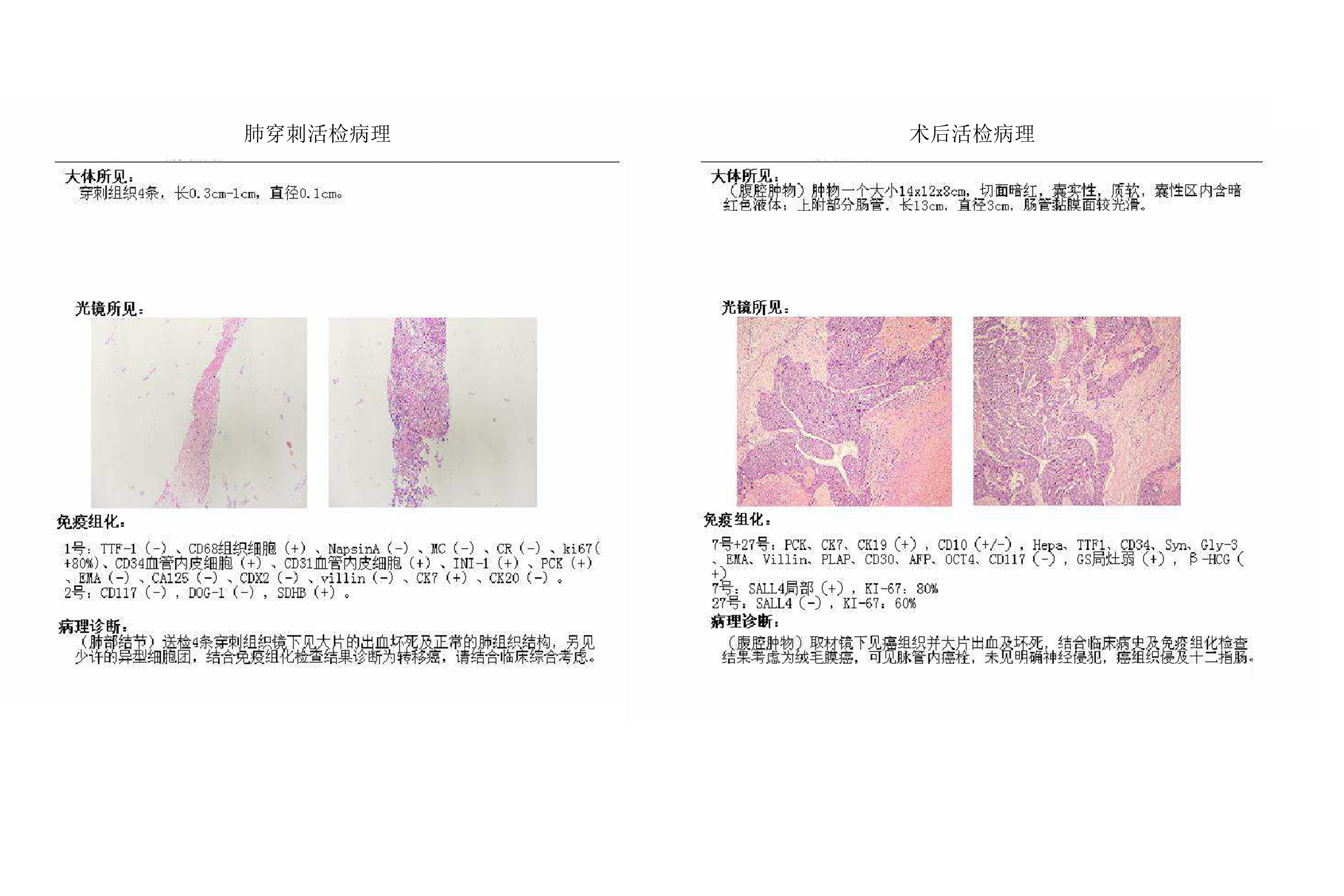

Supplement: Supplementary file 2 [file Image_2.tif]
